# Impact of social determinants and medical mistrust on parent-child HPV vaccination in economically disadvantaged communities: implications for cancer prevention

**DOI:** 10.3389/fonc.2024.1422839

**Published:** 2025-01-23

**Authors:** Marcelo M. Sleiman, Mary Rose Yockel, Mingqian Liu, Joanne Wendolowski, Lucile L. Adams-Campbell, Chiranjeev Dash, Lisa Carter-Bawa, Abraham Aragones, Sahana Arumani, Kenneth P. Tercyak

**Affiliations:** ^1^ Cancer Prevention and Control Program, Georgetown University-Lombardi Comprehensive Cancer Center, Washington, DC, United States; ^2^ Center for Discovery and Innovation, Hackensack Meridian Health-Hackensack University Medical Center, Hackensack, NJ, United States

**Keywords:** cancer prevention, cancer disparities, children, families, vaccination

## Abstract

**Introduction:**

Human papillomavirus (HPV) vaccination and intentions, their correlates, and barriers among age-eligible parents and their children living in very economically disadvantaged communities were assessed.

**Methods:**

Parents (N=198; 45% Black, 42% Latine, 57% educated <=high school [HS], 74% income <$60k annually) with children ages 10-17 from Washington, DC and Hackensack, NJ were intercepted at community events and surveyed.

**Results:**

Among age-eligible parents, 20% were vaccinated against HPV. Comparing vaccinated to unvaccinated parents, those who were non-white (OR=5.5, 95% CI=3.5, 9.4, p<0.001) and with unvaccinated children (OR=8.9, 95% CI=3.7, 23.3, p<0.001) were less likely to be vaccinated themselves. Among children, 37% were vaccinated. Unvaccinated children were more likely to have parents who were non-white (OR=2.7, 95% CI=2.6, 2.8, p<.01), with a <=HS education (OR=3.0, 95% CI=1.52, 6.25, p<.01), and were unvaccinated themselves (OR=10.2, 95% CI=4.01, 28.61, p<.001). Nearly two-thirds (63%) of parents with unvaccinated children expressed an intention to vaccinate within the next year: 48% confirmed receiving advice from a healthcare provider to do so. Common HPV vaccine barriers included lack of information (35%), safety concerns (16%), and perceptions of sexual inactivity (13%). An adjusted model revealed an interaction between parent education and medical mistrust (B=.35, SE=.13, 95% CI=0.09, 0.61, p<.01). For parents with <=HS education, when levels of provider trust were strong, they were more open to vaccinating their children.

**Conclusions:**

HPV vaccine prevalence was low among parents and children living in disadvantaged communities. Comprehensive education and intervention to build trust are warranted to prevent the spread of HPV-linked cancers and reduce cancer disparities.

## Introduction

Around 37,000 new cases of cancer annually in the United States can be linked to human papillomavirus (HPV), particularly types 16 and 18 (1–3). The most common HPV-associated cancers seen in females and males are cervical and oropharyngeal cancer, respectively (1). While the onset of HPV-associated cancers is prevalent, a disproportionate burden is carried by certain populations, such as those from historically-underserved and minority backgrounds. Rates of cervical cancer are highest among Latine and American Indian/Alaska Native women, and lowest among white women (4). Furthermore, Black women face high mortality from cervical cancer–at almost double the rate of white women (5). This disparity has been associated with low follow-up cancer screening rates (i.e. Pap testing) and delays to care (6). Socioeconomic divides also increase HPV-associated cancer disparities, with cervical and penile cancers being strongly linked to living in poverty (7). Yet, these outcomes are largely preventable: through vaccination and controllable with adequate resources for cancer screening and follow-up.

The quadrivalent HPV vaccine, 4vHPV, that was approved in 2006 for females aged 9-26 resulted in an 88% decrease in cervical cancer among vaccinated females below the age of 17 over the span of two decades (8, 9). Currently, the nine-valent (9vHPV) vaccine, which was recommended for females and males aged 9-26 in February 2015, is the only HPV vaccine available in the United States (10, 11). In June 2019, the Advisory Committee on Immunization Practices (ACIP) updated its recommendation such that those aged 27-45 years can receive the vaccine following a “shared clinical decision-making” model with their healthcare provider (12, 13). Health insurance plans cover the cost of HPV vaccines; the federally-supported Vaccines for Children program offering additional financial coverage for those who qualify. In the United States, the HPV vaccine is generally available without cost to eligible individuals under certain circumstances. Most private health insurance plans are required by the Affordable Care Act to cover the vaccine as part of preventive care, without any copayment or deductible, when administered by an in-network provider. For individuals who are un- or underinsured, the vaccine is often made available through programs such as Medicaid for people under the age of 21, and the Vaccines for Children program for those who meet criteria. Additionally, adults aged 19-45 years who lack insurance and meet income eligibility requirements can access the vaccine without cost through the vaccine manufacturer’s patient assistance program (14, 15).

Despite the existence of a vaccine for nearly two decades, full protection against HPV by way of a complete vaccination dose remains unacceptably low in the United States: only about 61% of males and 65% of female adolescents aged 13-17 completed a vaccination series in 2022 (the most recent date for which data are available) (16). In accordance with ACIP guidelines, it is recommended that children aged 14 and younger complete a two-dose HPV vaccination schedule, while those aged 15 and older are advised to receive a three-dose series to ensure optimal protection against HPV-related diseases (12). These include some forms of cancer that are eligible for screening by way of a pap smear, such as cervical cancer. In other countries with similar gross domestic product and human development levels to the United States, rates of HPV vaccine completion for females (for example) vary based on program: Germany (51%), Canada (86%), Singapore (89%), and Australia (80%). In Canada, it is believed that strong school-based vaccination efforts have partly led to its success. In Singapore, HPV vaccination rates are thought to be high due to this country’s well-resourced public health initiatives (including public health education), and its healthcare infrastructure (17).

Numerous barriers to HPV vaccination continue to be reported, however–many of which are suspected to be rooted in social determinants of health. This terminology refers to a full spectrum of economic, environmental, and other factors (such as conditions into which people are born) (18). Examples include unsafe housing and neighborhoods, inadequate public transportation, racism, discrimination and violence, lack of education and job opportunities, and polluted air and water. Healthcare accessibility and affordability may further shape families’ health outcomes. This may be typified by the divide between rural and urban communities. Adolescents in rural areas have vaccination rates at least 10 percentage points less than those in more urban areas (19). Conversely, in a study by Ramphul and colleagues, they reported that HPV vaccination differs by community income levels, such that lower-income areas have higher vaccination (20). This “reverse disparity” has been reported previously as well (21). Social determinants that deter vaccination among young adult women include having lower levels of formal education and being less proficient in English. For men, lacking accessible primary care is commonly cited (22). Uninsured status and not seeing a healthcare provider in the past six months also decreases vaccination–underscoring the impact that systemic healthcare barriers play in HPV vaccination, and the need for expanded coverage (22).

Populations particularly susceptible to encounter these barriers are those from lower income Black and Latine communities. Vaccination rates are sub-optimal in pediatric primary care environments that serve these populations: only 41% and 20% of female adolescents from under-resourced Black and Latine communities report initiating and completing the vaccination series, respectively (23). While data show that lower income minority teens may be more likely to initiate vaccination relative to those who are white and come from higher-income households, they are less likely to complete the vaccination series (i.e., 2 doses for those under age 15; 3 doses for those older than 15) (24, 25). These levels of nonadherence to a multi-dose regimen are associated with a majority of low-income Black and Latine adolescents not receiving healthcare provider advice to vaccinate as often as other groups (25). Moreover, their providers are less likely to discuss and document sexual activity, exacerbating vaccine non-uptake (23, 25). Apart from provider recommendations, parents of adolescents who are from lower-income backgrounds, and those who are Black and Latine, report lacking information about the HPV vaccine and the importance of having to return for multiple doses (26). Parents who are Black and from lower-income backgrounds are also more likely to consider the HPV vaccine to be “new”, potentially contributing to misinformation and mistrust in the vaccine’s safety and efficacy (26). Among mothers who are Latine with lower socioeconomic backgrounds, less assimilation into United States society is associated with decreased vaccine uptake (27). According to the Centers for Disease Control and Prevention’s 2023 data, the prevalence of a completed HPV vaccine series for males and females (combined) among youth who are Black/African American was 59%, and for Latine was 63.6%, and for those living below the poverty level was 60.7% (28). These data suggest that more attention to cultural differences, and fostering trust in providers in the healthcare system, may be essential in understanding HPV vaccination behaviors among minority populations and offer clues to cancer prevention efficacy.

Due to the preventable nature of this cancer burden, and the vaccination barriers experienced by lower income families who are Black and Latine, our study sought to better describe their experiences with HPV vaccination. We did this by surveying populations in the greater Washington, DC (4.3 million people, 31% Black) and Hackensack, NJ (2.2 million people, 33% Latine) Census Bureau regions, where pronounced cancer disparities are known to exist. Vaccine initiation rates among youth ages 13-17 in the greater DC region is 84.6% in females and 80.8% in males: across the state of NJ, average rates are 73.4% in females and 77.5% in males (28). What is not yet known, however, are HPV vaccination prevalence rates among children and their vaccine-eligible parents who are living in families subsisting below the federal poverty line, and for whom targeted and tailored interventions do not exist. According to the World Health Organization (WHO), individuals living below 60% of the regional median household income are considered to be at heightened risk of health disparities, highlighting the critical need for more focused health initiatives in these communities (29). In the United States, regions with median household incomes of $96,346 in New Jersey and $111,000 in Washington, DC illustrate the financial challenges faced by many communities, which can contribute to barriers in healthcare access and exacerbate health disparities (29). We anticipated that HPV vaccination would be even less among these populations who were lower income and minoritized (i.e., Black and Latine) than state-wide community prevalence estimates suggest, and despite a school vaccination mandate in DC, partly due to numerous social determinants of health and other access burdens that families living in these areas commonly experience. Reported herein are parent-child HPV vaccine initiation rates, factors contributing to its uptake, vaccine hesitancy and medical mistrust, and vaccination intentions, as well as reasons for not vaccinating.

## Methods

### Study design

Prior to initiating the study, project leaders met with the university cancer center’s catchment area-wide community advisory board members who provided direction and feedback on the research objectives for the entire community, including a shared focus on parent-child HPV vaccination, barriers, and hesitancy to prevent cancer. A draft of the survey was circulated to these members, and revisions were made based upon their input. Survey data were then collected online by the sponsoring university with cancer center campuses in both regions, and administered in 2022 and 2023 to a unique and verified non-random sample of parents from lower-income and minority backgrounds in different households residing in greater Washington, DC and Hackensack, NJ by community outreach and engagement staff members employed by the cancer center. The study was reviewed and approved by Georgetown University Medical Center’s and Hackensack University Medical Center’s institutional review boards.

### Study population

The eligibility criteria for participation in this study were as follows: (1) at least 18 years old, (2) the parent or caregiver of one or more children 10-17 years of age, (3) residing in the sponsoring university cancer center’s catchment area (in DC: Anne Arundel, Howard, Prince George’s, St. Mary’s, or Montgomery counties in MD; and in NJ: Bergen, Hudson, or Passaic counties). Recruitment strategies included distribution of printed study flyers with a scannable QR code at 41 events (12 in NJ and 29 in DC) including: community events, a supermarket, churches, and other public venues. Recruitment of participants was led by community outreach coordinators. Some participants were also recruited over the telephone as part of cancer prevention outreach and education activities. Following informed consent, on-site surveys were administered in a face-to-face manner and were either completed on participants’ personal mobile devices or a mobile device made available by the study to N=198 parents, who self-reported both their own and their child’s data: a gift card was awarded to acknowledge participants’ time and effort.

### Data collection instruments

#### Survey overview

The survey collected the sociodemographic background of parents (age, sex, gender, race, ethnicity, education, and income), their HPV vaccine awareness (whether the participant had ever heard of it or not), and their own HPV vaccination status, as well as that of their children. Parents were also asked to report on their total number of children, as well as the age and sex of their oldest child who fell between the ages of 10-17, and the number of doses of the HPV vaccine that child received. (Due to privacy concerns, the exact ages of some parents’ children were not given.) If the oldest child had not initiated HPV vaccination, the survey directed them to specify reasons for nonvaccinating. Other measures included receipt of educational materials from the child’s school about the HPV vaccine, receipt of a healthcare provider recommendation, level of trust in medical professionals and the HPV vaccine, and the behavioral intent to vaccinate this child within the next 12 months.

#### Parent vaccine eligibility

At the time of analysis, for the parent to be considered age-eligible for the HPV vaccine, it was necessary to calculate the age of every parent as of June 26, 2019, when the HPV vaccine was first approved for adults aged 27-45 by the Advisory Committee on Immunization Practices (12, 13). Based on our calculation, parents 46 years of age or older as of that date, would have been ineligible for HPV vaccination. Of the N=198 total parents, n=130 (66%) were deemed “age-eligible” for the HPV vaccine.

#### HPV vaccination initiation, prevalence, and intentions

Self-reported parent-child vaccination prevalence rates were reported: individuals were grouped into those receiving zero injections (including those who said they didn’t recall, to bias against the hypothesis) or at least one injection (i.e., 1-3 injections). Parents with unvaccinated children were prompted to select their top three reasons for this decision from a list of 25 possible reasons that were empirically derived from the literature (30, 31). The survey also asked parents with unvaccinated children to rate their vaccine intent in the next twelve months (responses were on a four-point Likert scale - “Not likely at all,” “Somewhat unlikely,” “Somewhat likely,” and “Very likely”).

#### Social determinants of health, medical mistrust, and HPV vaccine hesitancy

The following social determinants of health were treated as model covariates: age of parent, race, ethnicity, education level, household income; medical trust and vaccine hesitancy were also included. Parents rated how much they agreed with the statements that ‘medical professionals have their best interests at heart’ and that ‘vaccine benefits outweigh the risks’ (strength of agreement was ranked on a five-point Likert scale - “Strongly disagree,” “Disagree,” “Neither disagree nor agree,” “Agree,” “Strongly agree”). This medical trust item was adapted from the 7-item Hopkins Medical Mistrust Index, which measures someone’s mistrust of healthcare providers, organizations, and health systems (32). The item about vaccine risk was adapted from the 9-item Vaccine Hesitancy Scale, which measures a person’s level of vaccine hesitancy (33).

### Data analysis

Sociodemographic characteristics of parents were summarized using univariate statistics (see **Table 1**). Bivariate analyses were conducted to investigate associations among sociodemographic factors and HPV vaccination rate, medical mistrust, vaccine hesitancy, and vaccination intent; those with suggestive relationships at p<=.10 were considered further. Multivariate logistic regression was then utilized to examine adjusted associations across factors, including sociodemographics, parent vaccination, medical mistrust, and vaccine hesitancy, and their relationship to parent and children vaccination rates and intentions to vaccinate children. Regarding HPV vaccination barriers, we first generated descriptive statistics about parents’ vaccine hesitancy from an array of 25 empirically derived choices (30, 31). The data were then analyzed in rounds. In the initial round, three independent coders (M.M.S., M.R.Y., S.A.) thematically grouped each of the 25 possible reasons for vaccine hesitancy into higher-order categories: this resulted in 13 classes of vaccination hesitancy. In the next round, these 13 classes were further reduced by 15-30% across the coders: coder agreement was high at 81% and with excellent intercoder reliability. We then applied this final coding scheme to the available study data. The process resulted in five discrete codes, with an additional code used to capture more general/nuanced comments that were not otherwise represented. Coding discrepancies were resolved by consensus. The frequency of each code was then computed, indicating the number of times each code was applied to the dataset. These frequencies were then summarized to yield an overall count of the total number of coded responses, along with each code’s percentage of the total.

**Table 1:** Sociodemographic characteristics of parents and children (N=198).

	Mean	SD	n	%
Parents				
Age	42.57	11.87	179	90.4
Vaccine-eligible (based on age), Yes			130	72.6
Sex				
Male			26	13.1
Female			172	86.9
Gender				
Male			27	13.7
Female			165	83.8
Transgender			2	1.0
None of these			3	1.5
Race				
Black/African American			89	45.4
White			69	35.2
Other (including Asian American/Pacific Islander)			38	19.4
Ethnicity				
Latine			81	42.2
Non-Latine			111	57.8
Education				
High school diploma/GED and less			109	57.1
More than high school/GED			82	42.9
Annual income				
Less than 30,000			106	54.4
$30,000 to $59,999			38	19.5
$60,000 to $99,999			16	8.2
$100,000 or more			8	4.1
Decline to answer			27	13.8
Children				
Age	13.28	2.55	163	
Sex				
Male			92	47.7
Female			101	52.3

Parents’ vaccine eligibility was based on their age at the time of ACIP recommendation, and reported among those with complete age data. Within Latine ethnicity, 85.1% reported a household income under $60,000 annually. Of the N=198 parents surveyed, a total of N=163 (82%) reported on their children’s ages and N=35 (18%) did not.

### Data availability

The data generated in this study are not publicly available as the information could comprise patient privacy and consent.

## Results

### Sample characteristics

Demographic characteristics of the N=198 study participants are displayed in **Table 1**. Parents’ mean age was 42.6 years (SD=11.9), and a majority identified as female (83.8%), Black (45.4%) or Latine (42.2%), with a high school education/GED or less (57.1%), and an annual income of less than $60,000 (73.9%): falling below median household income for these geographic areas. Approximately the same number of parents were recruited from the greater Washington, DC (54.0%) and Hackensack, NJ (46.0%) metro areas. Based on the age data provided, children of these parents averaged 13.3 years (SD=2.6) and 52.3% were female.

### HPV vaccination prevalence

The prevalence rates of HPV vaccination for parents and their children are reported below, along with parents’ intention to vaccinate their children who had not yet received at least one dose of an HPV vaccine. We also describe the results of our content analysis, revealing major themes in vaccine hesitancy within the study population.

#### Parents

**Figure 1:**
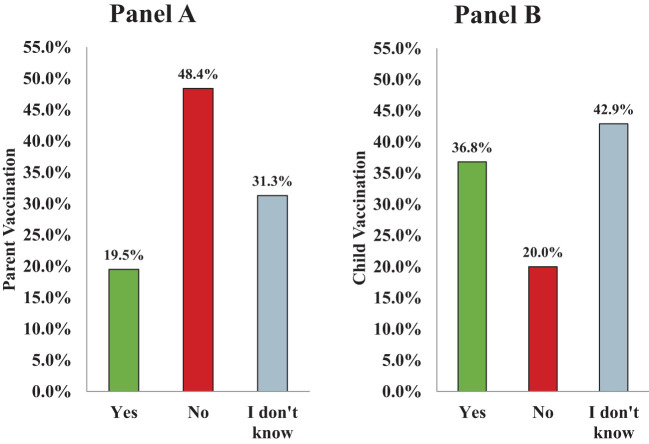
Prevalence of HPV vaccination among parents and their children. Parents were asked to self-report their own and their oldest child’s vaccination status. **(A)** shows that among parents deemed to be age-eligible for the vaccine, about 20% received at least one dose of the vaccine, 48% did not receive any doses of the vaccine, and 31% did not know their vaccination status. **(B)** shows that among the oldest children of parents surveyed, about 37% initiated vaccination, 20% were unvaccinated, and about 43% had an unknown vaccination status.

Among age-eligible parents, the HPV vaccination rate [adjusted for the introduction of HPV vaccination by birth cohort, according to the Advisory Committee on Immunization Practices (12, 13)] was 20%, with 48% reporting not being vaccinated, and an additional 31% unable to recall their own vaccination status (**Figure 1**). Bivariate analyses revealed that older parents (F=6.9, df=2, p<.01), those who were non-white (X2 = 9.1, df=4, p<.10), and those of Latine ethnicity (X2 = 5.6, df=2, p<.10) were the least likely to self-report being vaccinated against HPV. Additionally, parents who did not have their child vaccinated were significantly less likely to have self-reportedly received the HPV vaccine (X2 = 17.6, df=1, p<.01). Logistic regression analyses confirmed these associations: compared to parents who were vaccinated, non-white parents (OR=5.5, 95% CI=3.5, 9.4, p<0.001) and those without vaccinated children (OR=8.9, 95% CI=3.7, 23.3, p<0.001) were less likely to be vaccinated themselves.

#### Children

Among children of parents surveyed by self-report, 37% had received one or more doses of the HPV vaccine, 20% had not, and an additional 43% of parents could not recall their own child’s vaccination status. In bivariate analyses, children were less likely to be vaccinated if they had parents who were: non-white (X2 = 4.85, df=2, p<.10), with lower levels of formal education (X2 = 5.09, df=1, p<.05), living in lower-income households (X2 = 5.23, df=1, p<.05), and were unvaccinated themselves (X2 = 17.64, df=1, p<.001). Logistic regression revealed that, compared to children who were vaccinated, those who were not (or had parents who could not recall) were more likely to have parents who were non-white (OR=2.7, 95% CI=2.6, 2.8, p<.01), with a high school education or less (OR=3.0, 95% CI=1.52, 6.25, p<.01), and who were not vaccinated themselves (OR=10.2, 95% CI=4.01, 28.61, p<.001).

#### HPV vaccination intentions and barriers

**Figure 2:**
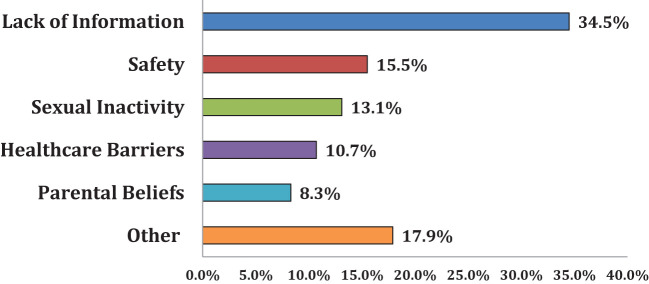
Barriers to HPV vaccination. Parents with unvaccinated children were asked to select their top three reasons for not vaccinating their child. Parents were presented with a list of 25 reasons that were thematically sorted into the six categories shown in the figure. The most cited reasons fell under the “lack of information” category (34.5%), followed by concerns of vaccine safety (15.5%) and low perceived need due to sexual inactivity (13.1%). “Lack of information” includes “I have never heard of the vaccine/unaware of it,” “I didn’t know it was for both males and females,” “don’t know enough about it/I need more information,” “I was unaware of HPV/did not know the vaccine was recommended to protect against HPV,” “I was unaware of where to go to have my child vaccinated” and “I have never heard of the vaccine/unaware of it”. “Vaccine Safety” includes “I don’t trust the HPV vaccine”, “Concerns about HPV vaccine safety or side effects”, and “My child has a chronic illness or was sick”. “Sexual Inactivity” includes “Child is not sexually active”. “Healthcare Barriers” includes “I have not taken my child to see their primary care provider recently”, “The HPV vaccine costs too much and/or is not covered by my health insurance”, “I had a bad experience with previous healthcare”, and “My child’s healthcare provider did not recommend.” “Parental Beliefs” includes “I don’t believe my child needs to be vaccinated against HPV (now/in the future)”, and “Because of religious reasons”. “Other” includes having plans to vaccinate, “immigration status”, the COVID-19 pandemic, “don’t know”, and “no school mandate.”.

Nearly two-thirds (63%) of parents with unvaccinated children expressed an intention to vaccinate them within the next year: 48% confirmed receiving advice from their healthcare provider to vaccinate their child, 22% were unadvised, and 30% could not recall the advice. As shown in **Figure 2**, major themes in parents’ HPV vaccine barriers included: lack of information (35%), concerns about vaccine safety (16%), perceptions of sexual inactivity (13%), access barriers (11%), and low perceived need (8%). Additionally, other reasons emerged that could not be as easily categorized. For example, the impact of family’s immigration status on seeking healthcare in the United States (34), the COVID-19 pandemic, and lack of a school-based mandate (see note to **Figure 2**).

Bivariate associations indicated that parents with both higher levels of medical mistrust (r=.24, p<.05) and vaccine hesitancy (r=.31, p<.01) were less likely to intend to vaccinate. In a multivariable model that tested parent education and medical mistrust as predictors of vaccine intention, and controlling for race and parental vaccination status, an interaction was observed between education and mistrust (B=.35, SE=.13, 95% CI=0.09, 0.61, p<.01). Specifically, for those with a high school education or less, when levels of provider trust were uniformly strong (i.e., 80% agreement that providers ‘have children’s best medical interests at heart’), parents’ vaccine intentions suggested that they were open to having their children vaccinated within the next 12 months.

## Discussion

This study, which focused on financially distressed and racially and ethnically diverse communities in DC and NJ, revealed remarkably low HPV vaccination prevalence among both vaccine-eligible parents (20%) and their children (37%). Importantly, and has not often been reported in the prior literature, approximately one-third of adults could not recall their own vaccination status, and about half could not recall their children’s. Reasons for this lack of awareness are unknown. On the one hand, it may reflect low levels of health literacy within this population, a lack of engagement with healthcare and/or healthcare providers, and/or a lack of contextual cues to adequately recall one’s own or one’s child’s vaccination history. On the other hand, it may be reflective of a larger and more systemic phenomena that deserves further study of how to support these parents in understanding their own and their children’s health. Regardless of its cause, the consequences remain the same: parents and children in these communities are underserved from a vaccination perspective, with missed opportunities to educate and inform about HPV vaccination and the delivery of this care to those most in need.

In this study, social determinants of health played a significant role in the findings because parents who identified as non-white, and had unvaccinated children, exhibited the lowest vaccination rates. Moreover, children of these parents who were unvaccinated and whose parents possessed lower levels of formal education, were less likely to have initiated an HPV vaccination series. Parental decisions to forego vaccination on their own and/or their child’s behalf are multifaceted, including: a lack of awareness about HPV and the vaccine (34.5%), uncertainty regarding vaccine safety (15.5%), and the perception that a child’s sexual activity status determines vaccination necessity (13.1%). Child vaccination rates within families from lower-income and minority backgrounds reveal influences of systemic barriers as well. It was expected that the child vaccination rate in these families would be lower than national averages. This likely arises from the presence of social determinants of health barriers, such as financial insecurity or unsafe living environments, that, unfortunately, deter access to high quality healthcare for these individuals (35, 36).

The intersecting nature of structural barriers and social determinants of health within these communities contributes to a concerning lack of access, exacerbating disparities in healthcare utilization. One consequence of this limited access is a decreased awareness of HPV and a subsequent underutilization of the vaccine as a method of primary cancer prevention. This trend is especially pronounced among populations facing the highest cancer mortality rates, further emphasizing the impact of these forces on their health outcomes (5, 37). The net result of inadequate access extends beyond the immediate implications for HPV vaccination. Notably, diminished awareness of HPV within these populations poses an ongoing public health challenge (38). A lack of knowledge about the virus and preventive measures also hamper efforts to curb the spread of HPV-related cancers that contribute to higher mortality rates within these already vulnerable communities (39).

Encouragingly, most surveyed parents with currently unvaccinated children expressed an intent to vaccinate them within the next year. Even among parents with lower levels of formal education, these data demonstrated how a trusting relationship with a healthcare provider would boost the likelihood that parents were receptive to vaccinating their sons and daughters over time, with a one-unit increase on the provider trust scale correlated with a 0.35 point increase in vaccination intent. This is important because of the well-established relationship between behavioral intention to vaccinate and HPV vaccination behavior that is posited by models of health behavior change (40, 41). The impact that intention to vaccinate has on vaccine uptake suggests that forming trusting relationships holds great potential to reduce cancer-related health disparities. Yet, parents’ formal education level should not determine which vulnerable groups are more equipped with the knowledge necessary to make informed choices about their own and their children’s healthcare.

From a policy perspective, the United States has already established national benchmarks for HPV vaccination as part of its Healthy People initiative (42). Unlike other countries around the world, the United States has maintained a multi-dose regimen of the HPV vaccine which, while providing full coverage, places additional burdens on families least likely to medially adhere to this recommendation (43, 44). Whether or not moving from a multidose to single dose regimen would eradicate HPV-linked cancers in the United States (as trends outside the United States suggest) remains to be seen (45, 46).

HPV vaccination has been marked by hesitancy since its introduction in 2006. Nearly a quarter of parents in the United States still harbor feelings of hesitancy toward the HPV vaccine for their children (47). Our own data suggest multiple barriers that families from under-resourced communities confront when vaccinating their children against HPV. These included how informed families are about HPV and the benefits that come from vaccination, concerns with vaccine safety, and their children’s sexual activity level/perceived ‘need’ to vaccinate. Importantly, our data focus on vulnerable populations—underscoring and magnifying these findings from a cancer prevention perspective (48–50). Although provider recommendation is consistently observed as a driver of children’s vaccination, our analysis revealed that this provider recommendation should be accompanied by a solid foundation of trust with that provider. When communities have been overlooked and marginalized, the cumulative effect of that disenfranchisement on their health and social well-being is especially pronounced. In the current climate of social change taking place across the country, there is a renewed optimism and opportunity to repair broken relationships with families from underrepresented backgrounds, especially families like those included in our study.

This study recognizes the importance of joining with individuals where they are in their readiness to vaccinate to promote health outcomes, especially for those from lower income and minority communities. Tailoring interventions to address the concerns and medical mistrust dynamics within these communities is likely essential for overcoming persistent hesitancy and increasing vaccine acceptance. This multilevel approach involves culturally and developmentally appropriate health communications, community engagement, and educational campaigns. To effect positive change on HPV vaccination rates, it is encouraging to look at successful interventions that have addressed similar challenges. For instance, an intervention involving emailed HPV vaccine reminders in both English and Spanish significantly increased vaccine initiation among Black, Hispanic, and Asian adolescents (51). These successes may provide a blueprint for how tailored interventions can effectively address discrepancies in vaccination rates in the United States. With respect to other countries, including those with health systems that differ from those found in the United States, it would be important to understand how these types of interventions would need to be adapted to not only account for individual influences, but also social and institutional factors affecting HPV vaccination rates in those settings.

### Limitations to study

Limitations of this study include the use of a non-random sampling technique. However, the purpose of our approach was to reach communities of color who have been historically excluded and underrepresented in research (52). Additionally, all survey data were self-reported by the parents without verification from a health record and some parents opted not to provide complete age data for their children. Therefore, the data may be incomplete and also susceptible to recall and response bias. However, inability to recall one’s own and one’s child’s HPV vaccination history has been reported previously, suggesting that patients may need reminders and better access to medical records to stay informed (53). The survey also contained brief and streamlined behavioral assessments and was only administered in English, which may have precluded greater insights into vaccination barriers and excluded monolingual Spanish and other language speakers from participating. Finally, the study’s sample size was modest to more sensitively detect interactive effects in the data. Larger and more well-powered studies would need to be conducted in the future to draw definitive conclusions about the observed relationships reported herein.

## Conclusions

Suboptimal prevalence of HPV vaccine uptake was observed among parents and children in lower-income communities and racial/ethnic minority groups in the regions studied. Recognizing these social determinants of health barriers is essential for informing targeted interventions. Strategies should include comprehensive education initiatives and facilitated program access to address disparities and improve vaccine acceptance within these populations to control their cancer burdens.

## Data Availability

The datasets presented in this article are not readily available because This is a behavioral dataset on a vulnerable minoritized population. Requests to access the datasets should be directed to habit@georgetown.edu.
